# O-GlcNAcylation mediates the control of cytosolic phosphoenolpyruvate carboxykinase activity via *Pgc1α*

**DOI:** 10.1371/journal.pone.0179988

**Published:** 2017-06-23

**Authors:** Pedro Latorre, Luis Varona, Carmen Burgos, José A. Carrodeguas, Pascual López-Buesa

**Affiliations:** 1 Departamento de Producción Animal y Ciencia de los Alimentos, Facultad de Veterinaria, Universidad de Zaragoza, Zaragoza, Spain; 2 Instituto de Biocomputación y Física de Sistemas Complejos (BIFI), BIFIIQFR (CSIC) Joint Unit, Universidad de Zaragoza, Zaragoza, Spain; 3 Departamento de Anatomía, Embriología y Genética, Universidad de Zaragoza, Zaragoza, Spain; 4 Instituto Agroalimentario de Aragón (IA2), Zaragoza, Spain; 5 Departamento de Bioquímica y Biología Molecular y Celular, Facultad de Ciencias, Universidad de Zaragoza, Zaragoza, Spain; 6 IIS Aragón, Zaragoza, Spain; Universitat de Lleida, SPAIN

## Abstract

PGC1*α* is a coactivator of many transcription factors and cytosolic phosphoenolpyruvate carboxykinase (PCK1) is a key enzyme for gluconeogenesis. PGC1*α* interacts with the transcription factor PPARγ to stimulate PCK1 expression and thus *de novo* glucose synthesis. These proteins are not only important for central energy metabolism but also for supplying intermediates for other metabolic pathways, including lipidogenesis and protein synthesis and might therefore be important factors in the ethiopathogenesis of metabolic disorders like diabetes but also in other pathologies like cancer. Since polymorphisms in these proteins have been related to some phenotypic traits in animals like pigs and PGC1α G482S polymorphism increases fat deposition in humans, we have investigated the molecular basis of such effects focusing on a commonly studied polymorphism in pig Pgc1*α*, which changes a cysteine at position 430 (WT) of the protein to a serine (C430S). Biochemical analyses show that Pgc1*α* WT stimulates higher expression of human PCK1 in HEK293T and HepG2 cells. Paradoxically, Pgc1α WT is less stable than Pgc1α p.C430S in HEK293T cells. However, the study of different post-translational modifications shows a higher O-GlcNAcylation level of Pgc1α p.C430S. This higher O-GlcNAcylation level significantly decreases the interaction between Pgc1α and PPARγ demonstrating the importance of post-translational glycosylation of PGC1α in the regulation of PCK1 activity. This, furthermore, could explain at least in part the observed epistatic effects between PGC1α and PCK1 in pigs.

## Introduction

PGC1α was first discovered as a cold-inducible protein involved in muscle thermogenesis [1]. To coordinate metabolic responses PGC1α interacts with a large number of transcription factors, including PPARγ [1], stimulating the expression of gluconeogenic enzymes such as cytosolic phosphoenolpyruvate carboxykinase (PCK1). PCK1 catalyzes the reversible reaction that decarboxylates OAA (oxaloacetic acid) to obtain PEP (phosphoenolpyruvate). PEP is a precursor of several biomolecules, being a key regulatory enzyme of gluconeogenesis, glyceroneogenesis and other metabolic pathways [2]. We have recently reported the effects of pig Pck1 c.A2456C substitution on fat distribution in pigs [3]. The c.A2456C substitution produces a Met139Leu substitution in Pck1 that modifies the kinetic properties and protein stability *in vivo* and *in vitro*. This SNP is also associated, among others, with a decrease in backfat thickness (BT), a change in water-holding capacity and an increase in intramuscular fat content (IMF), traits related to fat metabolism and meat quality.

PGC1α regulates not only metabolic responses, it also contributes to mitochondrial biogenesis [4], interacts with proteins with histone acetyltransferase activity [5] and determines the type of muscle fiber [6]. In humans, the G482S variant of PGC1α has been shown to modify fat metabolism in hepatocytes [7]. Several polymorphisms have been described in pig *Pgc1α* [8–10]. The most studied one [8], c.T1378A, produces a single amino acid change from cysteine to serine at position 430 (Cys430Ser). The reports about its phenotypic consequences are conflicting, ranging from the finding of no effects [9,10] to changes in muscle pH values [11] or intramuscular fat content [12].

Given the role of PGC1α in regulating PCK1, we hypothesized that the Cys430Ser substitution in Pgc1α modulates PCK1 activity. We have studied whether post-translational modifications of PGC1α could influence expression of PCK1, focusing on differences in glycosylation between Pgc1α wild type (WT) and p.C430S proteins. We have found that although the WT variant is less stable than the p.C430S one, the former is responsible for higher expression levels of PCK1. Higher O-GlcNAcylation of p.C430S decreases its interaction with PPARγ, which explains the observed effect. Furthermore, this molecular mechanism can explain the potential epistatic effect observed between *Pgc1α* and *Pck1*.

## Materials and methods

### Cloning

Human DYK tagged O-GlcNAc transferase (OGT, OHu54262) and pig Pgc1α-myc (OSe00030D; p.C430S) clones were obtained from GenScript. Pgc1α was subcloned into pEGFP-N1 using BamHI and XhoI restriction enzymes. Site-directed mutagenesis was performed using *Pfu* Ultra II HS DNA polymerase (Agilent) and *DpnI* (Fermentas). The following primers were used to create p.430C variant in pig Pgc1α (PGC1aSer430Cys-F 5’-ccacagactcagaccagtgctacctgaccgagacgtcggag-3’ and PGC1aSer430Cys-R 5’-ctccgacgtctcggtcaggtagcactggtctgagtctgtgg-3’). The stop codon was eliminated to overexpress Pgc1α-myc-GFP using PGC1aEGFP-F 5-catctcagaagaggatctgttggatccaccggtcgccacc-3 and PGC1aEGFP-R 5-ggtggcgaccggtggatccaacagatcctcttctgagatg-3.

### Quantitative PCR assays

To determine differences in Pgc1α activity, mRNA levels of endogenous PCK1 were measured. HEK293T cells (4x10^4^) were grown on 96-well plates using complete DMEM (DMEM supplemented with 10% fetal bovine serum, 2 mM L-glutamine, 0.1 mg/mL streptomycin and 100 U/mL penicillin). After 24 hours, cells were transfected using GeneJuice (Novagen) following manufacturer's instructions. 24 hours later, mRNA was extracted and cDNA was obtained using Cells to Ct 1-Step Taqman kit (Thermo Fisher Scientific). PCK1 cDNA was amplified using a human PCK1 probe set (Thermo Fisher Scientific) in an Mx3005P qPCR System (Agilent). GAPDH was used as control. PCK1 was quantified as $\frac{1}{2^{\Delta Ct}}$; where Δ_Ct_ is Pck1_Ct_ − GAPDH_Ct_ from each sample.

### Endogenous PCK1 detection

HEK293T cells (ATCC, CRL-3216) (7.5x10^5^ per well) were grown on 6-well plates and treated as described above. HepG2 (1x10^6^) cells were maintained in MEM supplemented with 10% FBS, grown on 6-well plates and reverse transfected using GeneJuice: trypsinized cells were directly plated on the transfection mixture, prepared following manufacturer’s instructions. 24 hours post-transfection cells were lysed in PBS containing 1% NP-40 (Sigma) and a protease inhibitor cocktail (1 μM PMSF, 10 μM benzamidine and 0.5 μM leupeptin). Samples were boiled in SDS loading buffer and loaded into a 10% polyacrylamide gel. Proteins were transferred onto a PVDF membrane and blocked with PBS containing 0.5% Tween20 (Sigma) and 10% skimmed milk for 1 h. The membrane was incubated overnight at 4^°^C with Anti-PCK1 (Abcam Ab28455, 1:500) and anti-actin (Sigma A2066 1:20000) and then washed and incubated with secondary HRP-conjugated anti-rabbit antibody (Millipore 12–348, 1:10000) for 1h at RT. Western blots were visualized by ECL (Millipore).

### Glucose production assay

To analyze metabolic differences between the p.430C and p.430S variants, we measured the ability of HEK293T cells to produce glucose when overexpressing each variant. Glucose production was measured as previously described [13]. Cells were grown on 24-well plates. 24 hours after transfection, medium was removed, cells were washed once with PBS and 0.5 mL of glucose production medium was added (DMEM without glucose and phenol red supplemented with 2 mM sodium pyruvate and 20 mM sodium lactate). After 6 hours, half of the medium was collected and a colorimetric assay was performed (Sigma, GAGO20). Data were normalized to the total protein content from the whole cell lysates measured by Bradford assays.

### Subcellular localization of Pgc1α variants

Transfected HEK293T cells were grown on 10 mm round cover slides in a 24-well dish plate and incubated in complete DMEM supplemented with 2 μM Hoechst 33342 (Invitrogen) for 25 minutes to stain nuclei. Then, cells were fixed in 3.7% formaldehyde at RT for 20 minutes, washed with PBS three times and once with water. Samples were mounted on microscope slides using ProLong Gold Antifade Mountant (Thermo Fisher) and observed using a DMI6000 Leica fluorescent microscope.

### Protein stability in HEK293T cells

HEK293T cells (7.5x10^5^) were grown on 6-well plates and treated and transfected as described above. Medium was replaced with complete DMEM supplemented with 150 μg/mL cycloheximide (Sigma) to inhibit protein synthesis. Cells were collected at different time points (0, 5, 10, 20, 40 and 60 minutes) in PBS supplemented with the aforementioned protease inhibitor cocktail and 10 μM of proteasome inhibitor MG132 (Sigma) to avoid rapid degradation of Pgc1α. Cells were pelleted and lysed in SDS-PAGE loading buffer. Proteins were separated in 8% polyacrylamide gels and transferred onto a PVDF membrane. Blocked membranes were incubated with anti-myc (Invitrogen R950-25, 1:5000) and anti-actin and visualized as described previously. Western blots were analyzed using ImageJ (NIH).

### Immunoprecipitation and co-immunoprecipitation

To determine differences in post-translational modifications (PTMs), each variant was immunoprecipitated from mammalian cells. HEK293T cells were grown on 6-well plates, co-transfected with or without OGT and either Pgc1α WT or Pgc1α p.C430S. When specified, cells were incubated in DMEM supplemented with PUGNAc at 100 μ3ϕoρ 18η to inhibit O-GlcNAcase. Cells were lysed in 50 mM TRIS, pH 8, 150 mM NaCl, 1% NP-40, 0.1 mM EDTA, 2 mM nicotinamide, 10 mM NaF and protease inhibitor cocktail. Clarified cell lysates were incubated with 5 μg of anti-myc (Invitrogen 9E10 clone) at 4^°^C overnight. Pgc1α was immunoprecipitated using 25 μL of Dynabeads (Invitrogen) at 4^°^C for 2 hours. Non-transfected cells were used as control. Samples were boiled in SDS-PAGE loading buffer, run in 8% gels and transferred to PVDF membranes. These membranes were incubated with anti-myc, anti-O-GlcNAc (Abcam, Ab2739, 1:5000), anti-acetyl-lysine (Cell Signal, 9441, 1:1000), anti-PhosphoSer/Thr (Abcam, Ab17464, 1:1000) and anti-PPARγ (Santa Cruz, sc-7196, 1:500). Clean blot HRP detection kit (Thermo Fisher Scientific) was used to detect PPARγ from immunoprecipitates. Band density was analyzed using ImageJ.

### Statistical analysis

Data normality was analyzed using Shapiro-Wilk test. For glucose, qPCR and PCK1 western blotting, ANOVA tests were performed following Tukey's post-*hoc* tests. In the case of cycloheximide, immunoprecipitation and co-immunoprecipitation assays, Welch's two sample *t*-tests were performed.

### Isolation of genomic DNA

Genomic DNA was extracted from tails of Duroc x Landrace/Large White piglets. DNA was extracted using the Realpure genomic DNA extraction kit (Durviz) following the manufacturer's instructions. DNA concentration in the extract was quantified using a NanoDrop 1000 spectrophotometer (Thermo Fisher Scientific).

### Phenotypic recording and analysis of the *Pgc1α* c.T1378A and *Pck1* c.A2456C genotypes

We used a total of 202 pigs that had been phenotypically characterized and also genotyped with respect to the c.A2456C SNP in Pck1 in a previous work [3]. DNA amplification was carried out with AccuPrime^TM^Taq DNA Polymerase High Fidelity (Invitrogen) using the primers described by Kunej et al. [8] to amplify the *Pgc1α* SNP region. The PCR was performed in an UNO-Thermoblock apparatus (Biometra). The *Pgc1α* PCR product was purified using the NucleoSpin Extract II kit (Macherey-Nagel) following manufacturer′s instructions, quantified using a NanoDrop 1000 spectrophotometer and sequenced at Sistemas Genómicos (Paterna, Valencia, Spain) to identify the *Pgc1α* genotype.

### Epistatic analysis

The statistical analysis for detection of additive and additive x additive epistatic analysis was performed with a linear model under the Bayesian framework. The model of analysis was:

y = wb + Xβ + Zu + Tp + λ_Pgc1α_a_Pgc1α_ + λ_Pck1_a_Pck1_ + λ_axa_i_axa_ + e, where y is the vector of phenotypic data, b is the covariate with live weight at slaughter, β is the vector of batch effects, u is the vector of polygenic additive genetic effects, p is the vector of litter effects, a_Pgc1α_, a_Pck1_ and are the additive effects of *Pgc1α*, *Pck1*, respectively, i_axa_ is the additive x additive epistatic interaction effect and e is the vector of residual effects. Further, w is the vector of live weights at slaughter, X, Z and T are the corresponding incidence matrices that links systematic and polygenic additive effects with data, λ_Pgc1a_, λ_Pck1_ and λ_axa_ are the vectors of the additive and additive x additive coefficients calculated with the orthogonal approach proposed by Álvarez-Castro and Carlborg [14]. We assumed bounded uniform distributions for the covariate (b), systematic effects (β) and the additive and additive x additive effects (a_Pgc1α_, a_Pck1_ and i_axa_). Further, the following multivariate prior Gaussian distributions were assumed for polygenic and litter effects: $U∼N(0,A\sigma_{a}^{2})$ and $p∼N(0,I\sigma_{p}^{2})$, where A is the numerator relationship matrix calculated from the pedigree information and I is the identity matrix. Finally, prior distributions for variance components ($\sigma_{a}^{2}$, $\sigma_{p}^{2}$ and $\sigma_{e}^{2}$) were bounded uniform. The analysis was performed using a Gibbs Sampler [15] using a single chain of 100,000 iterations after discarding the first 10,000 for the calculation of the posterior distributions. Relevance of additive or additive x additive effects was determined when the posterior distribution had a probability over (or below) zero greater than 0.9 or below 0.1. Other epistatic interactions (additive x dominant, dominant x additive and dominant x dominant) were not presented because of the scarcity of available information (the low number of animals and underrepresented haplotypes) to obtain appropriate posterior estimates.

## Results

### Pgc1α C430S substitution decreases PCK1 mRNA and protein levels and glucose production in HEK293T cells

**Fig 1:**
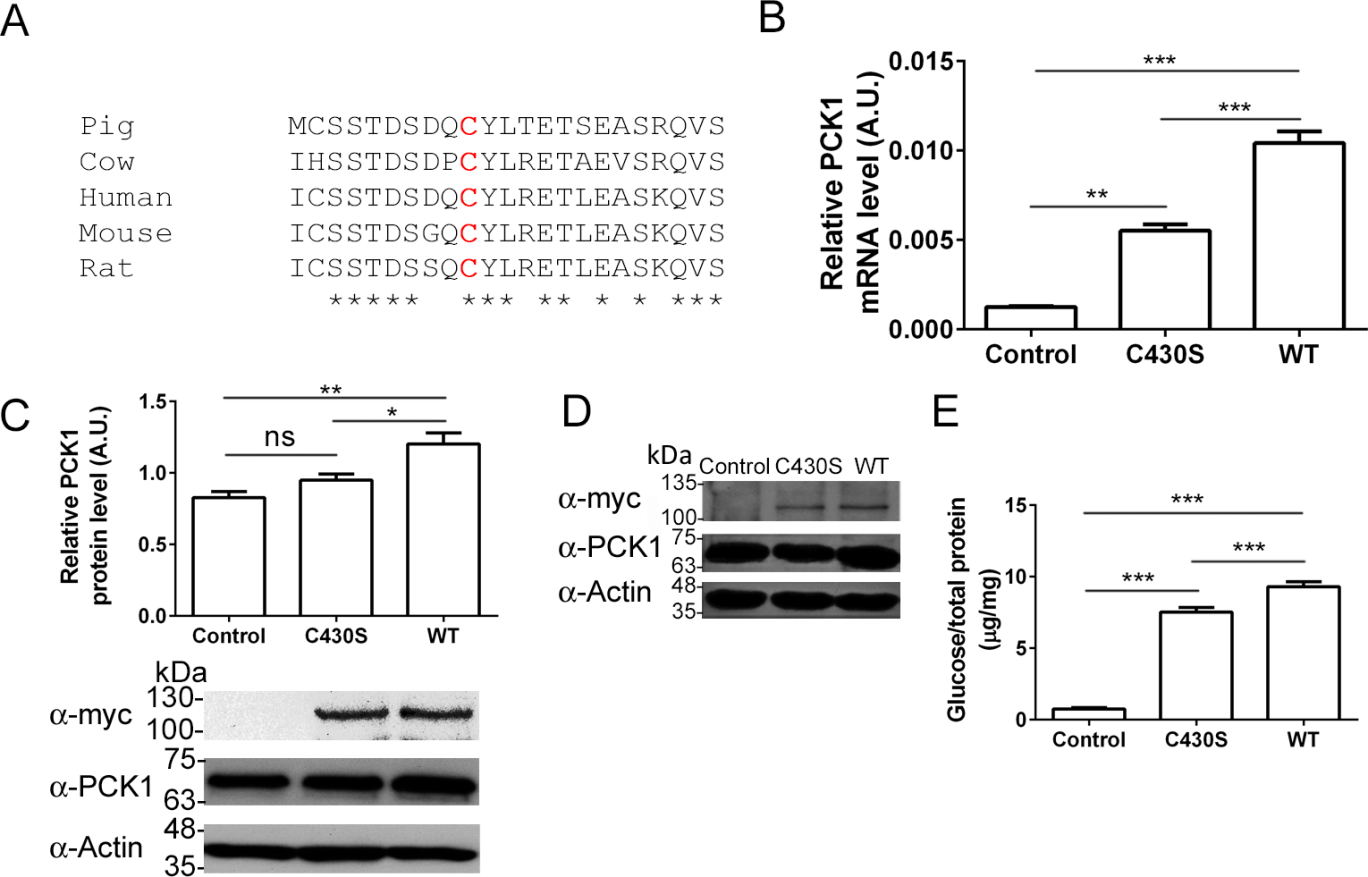
Cys430Ser substitution affects Pgc1α transcriptional activity. (A) As can be seen in the alignment, pig cysteine 430 (red bold) is well conserved among species and it also lies in a serine/threonine rich region. (B) mRNA levels of endogenous PCK1. HEK293T cells were transfected with either Pgc1α p.C430S or wild type (WT) and after 24h total mRNA was extracted and quantified using qPCR in three individual assays, using o mock transfected cells. Pgc1α WT produces more PCK1 mRNA than Pgc1α p.C430S. Shapiro-Wilk test was performed to check data normality. This experiment was repeated three times with three technical replicates each. ANOVA test showed significant differences between groups (n = 3; p = 1.3x10^-5^) and Tukey post-*hoc* test was performed to find differences among groups. (C) Pgc1α WT overexpression leads to a higher expression of PCK1 in HEK293T cells, as detected by Western blot. The experiment was repeated three times. The blots shown belong to a representative experiment. Bands were analyzed using ImageJ. Cells were treated and data was analyzed as described (n = 3; ANOVA, p = 0.0027). (D) Same experiment as (C) performed in HepG2 cells. (E) Cells overexpressing either Pgc1α p.C430S or WT were incubated in a free-glucose medium. After 6 hours, half of the medium was collected and glucose was determined using a colorimetric assay. Data were normalized to total cell protein content. Pgc1α p.C430S produces more glucose (19%) than Pgc1α WT. Three independent assays were carried out. Data was analyzed as described (n = 3; ANOVA, p = 3.3x10^-8^). In all cases, control cells were those transfected with empty vector. Asterisks indicate * p< 0.05, ** p< 0.01 and *** p< 0.001. C430S: Pgc1α p.C430S and WT: Pgc1α wild type.

Cysteine 430 is conserved among species (Fig 1A). We examined in HEK293T cells if pig Pgc1α p.C430S substitution could affect the expression of endogenous human PCK1. Fig 1B shows that this was indeed the case and that Pgc1α WT induced higher levels (2.4 fold) of PCK1 transcription than Pgc1α p.C430S, as measured by mRNA quantification using qPCR (Fig 1B). The amount of PCK1 protein detected by Western blotting in these experiments was also significantly larger when overexpressing Pgc1α WT than Pgc1α p.C430S in HEK293T and HepG2 cells (Fig 1C and 1D), although only by 20%. To determine if these differences in PCK1 protein content had a translation into metabolic performance of HEK293T cells, we analyzed glucose production in cells overexpressing each Pgc1α variant. It has to be remembered here that PCK1 is one of the main regulators of gluconeogenesis. Overexpression of both Pgc1α variants substantially increased glucose production compared to controls transfected with empty vectors, but cells overexpressing Pgc1α WT synthetized 19% more glucose (9.3±0.3 μg glucose/mg total protein) than those overexpressing Pgc1α p.C430S (7.5±0.3 μg/mg) (Fig 1E). This difference in glucose production matches nicely the difference in PCK1 protein amount found when expressing both Pgc1α variants.

### Stability and subcellular localization of Pgc1α variants

**Fig 2:**
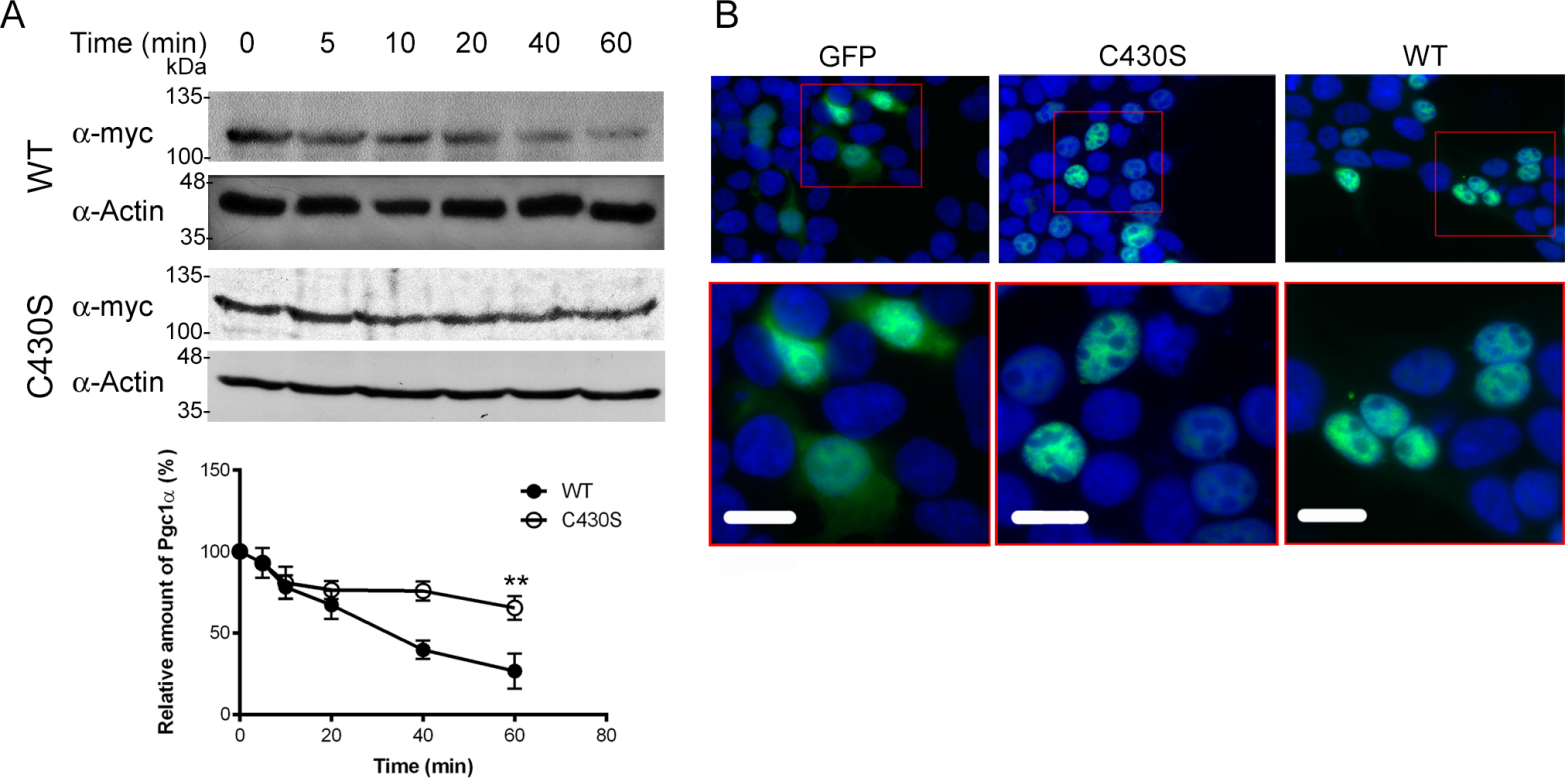
Cys430Ser substitution in Pgc1α affects protein stability but not subcellular localization. (A) Protein synthesis was inhibited with cycloheximide to look for differences in protein stability. As can be seen, Pgc1α WT has a shorter half-life. After 60 minutes, this difference is statistically significant (n = 3, p = 0.0095). Image shows a representative western blot of three independent experiments. (B) Subcellular localization of Pgc1α-GFP (green) in HEK293T cells. Hoechst 33342 was used to stain cell nuclei (blue). Overlays of images of green and blue channels are shown. Magnifications of red squares in upper panels are presented in lower panels. White line represents 10 μm. Transfected cells with empty vector show GFP in the cytosol and nucleus. In the case of both Pgc1α p.C430S and WT, they are located only in the nucleus. ** p< 0.01. C430S: Pgc1α p.C430S and WT: Pgc1α wild type.

The metabolic effects of any protein can be modified not only by increasing the amount of the protein or changing its functionality, but also by a change in protein stability. To investigate if the C430S substitution affected Pgc1α stability we analyzed Pgc1α content in HEK293T cells by Western blotting after cycloheximide addition. Cycloheximide blocks protein synthesis and allows following protein decay at different time points. Fig 2A shows that both Pgc1α variants significantly differ in stability: 60 minutes after cycloheximide addition, the amount of Pgc1α WT was reduced down to 26.7±10.8 (%) of the initial protein amount whereas that of Pgc1α p.C430S was only reduced down to 65.4±7.3 (%). This difference was statistically significant (n = 3, p = 0.0095). To rule out the possibility that the substitution could change the subcellular location of Pgc1α and modify in that way its stability, we analyzed the cellular location of GFP-tagged Pgc1α variants. As shown in Fig 2B the subcellular localization was not altered since both variants were localized in the nucleus.

### The effects of C430S substitution on post-translational modifications of Pgc1α

**Fig 3:**
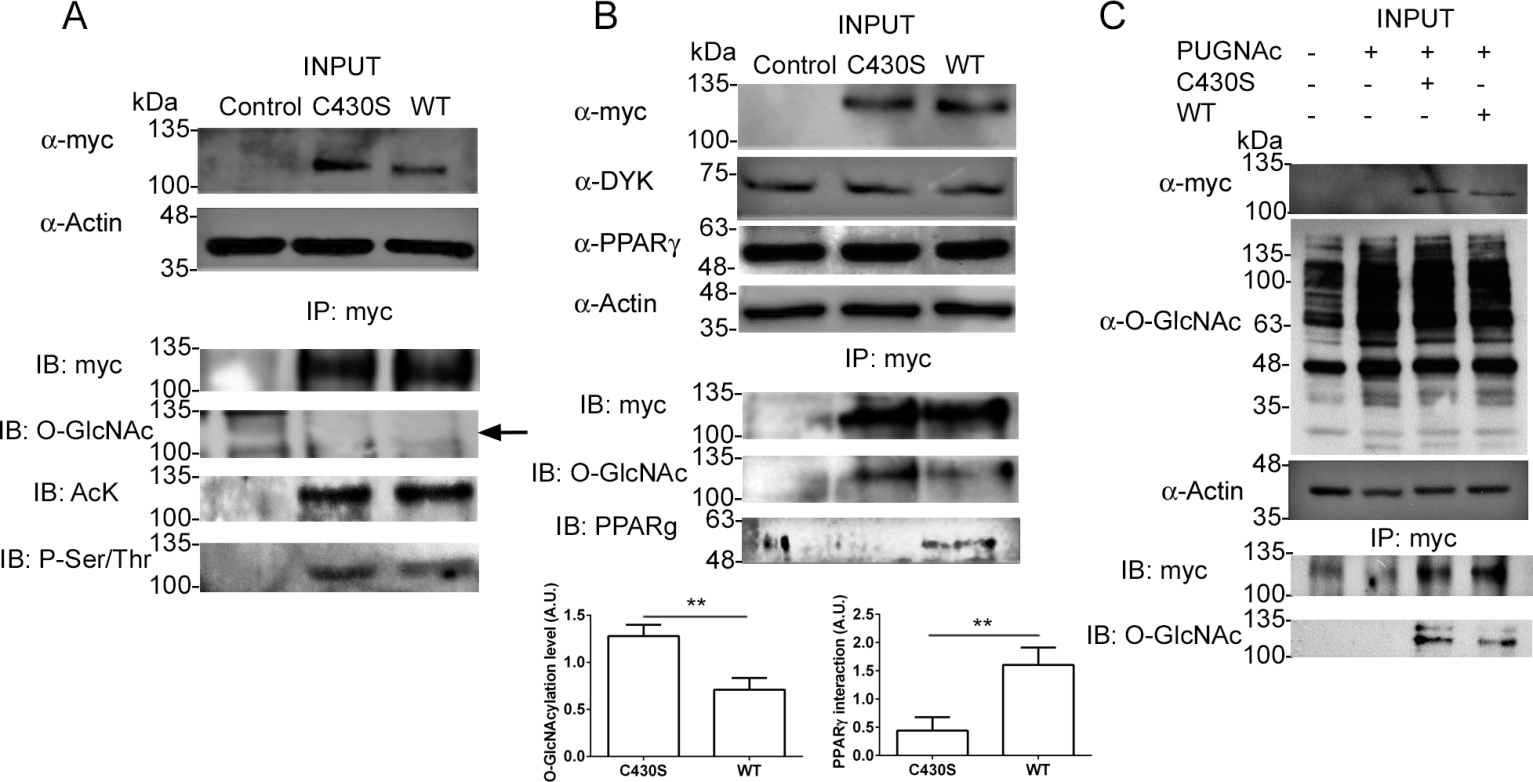
Cys430Ser substitution modifies O-GlcNAcylation level of Pgc1α and affects the interaction with PPARγ. (A) HEK293T cells overexpressing each Pgc1α variant were lysed and Pgc1α was immunoprecipitated using anti-myc. Afterwards, PTMs (glycosylation -O-GlcNAc-, lysine acetylation—AcK- and serine/threonine phosphorylation—P-Ser/Thr-) were studied and no differences were observed between the two Pgc1α isoforms (IP, immunoprecipitation; IB, immunoblotting). In fact, O-GlcNAcylation was too low to be detected under our conditions. (B) HEK293T cells were co-transfected with a DYK-tagged OGT-expressing plasmid and detected with an anti-DYK tag antibody (α-DYK). No differences are observed in the levels of acetylation or phosphorylation but Pgc1α p.C430S shows a higher level of O-GlcNAcylation than Pgc1α WT (n = 3, p = 0.0052). However, detection of PPARγ from immunoprecipitates shows that this protein has stronger interaction with Pgc1α WT (n = 3, p = 0.0079). These differences in O-GlcNAcylation and PPARγ interaction are graphically represented. ** p< 0.01. C430S: Pgc1α p.C430S; WT = Pgc1α wild type. (C) Cells were incubated in the presence of PUGNAc, a O-GlcNAcase inhibitor, for 16h and the levels of each Pgc1α variant were detected by western blotting with anti-myc and total protein O-GlcNAcylation was detected with anti-OGlc-NAc to show the effects of OGT inhibition. Then each Pgc1 variant was immunoprecipitated with anti-myc and detected with anti-myc and with anti-O-Glc-NAc. The lower panel shows a slightly lower level of O-GlcNAcylation of the WT protein with respect to p.C430S. Note that upon detection of immunoprecipitated Pgc1α with anti-myc a background signal is observed also in lanes corresponding to cells that do not express myc-tagged Pgc1α. This assay was carried out once with three technical replicates as an alternative to the experiment shown in Fig 3B.

The larger stability of Pgc1α p.C430S could, at least in theory, counteract its lower capacity to stimulate PCK1 synthesis. We analyzed therefore additional biochemical properties of Pgc1α that could be affected by the substitution and that could help to clarify the interaction between *Pgc1α* and *Pck1*. As Pgc1α has been shown to undergo several types of post-translational modifications (PTMs), such as phosphorylation, acetylation or O-GlcNAcylation, we decided to study whether the C430S substitution affected the ability of Pgc1α to undergo these covalent modifications. To do that, we immunoprecipitated both Pgc1α variants with anti-myc antibody and analyzed by Western blot with different specific antibodies their level of phosphorylation, acetylation or O-GlcNAcylation (Fig 3A). No difference was found between Pgc1α variants; indeed, no signal at all of O-GlcNAc was detected. We were somehow surprised because we did not detect any signal of O-GlcNAcylation in our experiments as this modification has been convincingly shown to modify Pgc1α stability [16], the property we were looking for. This led us to follow two different approaches, on one hand cells were co-transfected with both Pgc1α and OGT (Fig 3B), the enzyme that catalyzes O-GlcNAcylation, and on the other hand cells were treated with PUGNAc (Fig 3C), an O-GlcNAcase inhibitor. Fig 3B and 3C show that both cotransfection of Pgc1α and OGT and PUGNAc treatment led to detection of O-GlcNAc in both Pgc1α variants and that the level of O-GlcNAcylation was significantly lower in Pgc1α WT than that in Pgc1α p.C430S.

### The effect of O-GlcNAc level on Pgc1α-PPARγ interaction

PGC1α interacts with PPARγ to stimulate PCK1 expression [17,18]. Since we had found that the C430S variant affected PCK1 transcription (Fig 1), we decided to examine if the different level of O-GlcNAc of 430S and 430C could influence the ability of these variants to interact with PPARγ and affect in that way the expression of PCK1. Overexpressed Pgc1α variants, showing different levels of O-GlcNAcylation (Fig 3B), were immunoprecipitated using an anti-myc antibody and PPARγ was analyzed in the immunoprecipitates using a specific antibody. Fig 3B shows that Pgc1α p.C430S interaction with PPARγ was significantly diminished in comparison to that of Pgc1α WT. Thus, it can be reasonably proposed that this fact will contribute to an increase in PCK1 expression by Pgc1α WT mediated by PPARγ.

### Effects of PGC1α C430S substitution on phenotypic traits in pigs

We have previously reported [3], the influence of a PCK1 SNP (c.A2456C, M139L), in some phenotypic traits in pigs, and related it to differences in enzyme kinetics of both variants at the molecular level. This, together with the findings reported above, led us to analyze the influence of the C430S substitution in phenotypic traits in pigs and also a possible epistatic interaction between *Pck1* and *Pgc1α*.

Table 1 shows the Bayesian analysis of the influence of PGC1α c.T1387A (C430S) on conformational and meat quality traits on a previously described pig population [3]. For comparative purposes we present also the published effects of *Pck1* SNP on those traits in the same experimental group. S1 Table shows the same analysis for all studied traits. Genotypic frequencies for *Pgc1α* in this population were 29.8% AA, 56.1% AT and 14.1% TT. Only P values above 0.9 or below 0.1 were considered relevant for the additive and epistatic effects. None, but one, of the Bayesian P values of any traits were close to 0 or to 1 suggesting that the SNP in *Pgc1α* has no direct influence on those traits (Table 1).

**Table 1:** Posterior mean estimate and Bayesian posterior probability of the additive and additive x additive epistatic interaction effect between *Pgc1α* c.T1378A and *Pck1* c.A2456C.

Trait	Raw mean (SD)	A_1_	P	A_2_	P	A_1_xA_2_	P
**Backfat thickness**	19.85 (5.78)			0.873 (0.582)	0.067	-1.626 (0.744)	0.986
**Loin (%)**	5.73 (0.67)						
**Tenderloin (%)**	0.77 (0.10)	0.014 (0.010)	0.085				
**Ham (%)**	25.86 (1.27)					0.396 (0.179)	0.013
**Shoulder (%)**	15.18 (0.79)			-0.176 (0.089)	0.976	0.247 (0.118)	0.018
**Belly (%)**	6.63 (1.23)					-0.337 (0.172)	0.975
**Lard (%)**	12.23 (1.86)					-0.486 (0.291)	0.953
**Ribs (%)**	6.77 (0.90)					-0.353 (0.146)	0.992
**Skin and subcutaneus fat in ham (%)**	19.17 (3.64)					-0.826 (0.542)	0.936
**Whole ham muscle mass (%)**	64.96 (3.55)			-0.541 (0.383)	0.921		
**Ham bones (%)**	15.86 (1.99)						
**pH after 24h**	5.64 (0.15)						
**pH after 45min**	6.34 (0.23)						
**Color L**	50.46 (2.98)						
**Color a**	6.44 (1.26)						
**Color b**	1.54 (0.76)					0.209 (0.127)	0.050
**Drip loss after 1 day**	4.27 (1.91)			0.486 (0.214)	0.012	0.603 (0.277)	0.015
**Drip loss after 3 days**	6.56 (2.38)			0.754 (0.286)	0.004	0.633 (0.351)	0.036
**Drip loss after 7 days**	8.10 (2.49)			0.770 (0.303)	0.006	0.900 (0.409)	0.014

SD = Standard deviation; P = Bayesian probability above zero; A_1_ = Additive effect of *Pgc1α* and A_2_ = Additive effect of *Pck1*. Only data whose P is above 0.9 or below 0.1 are presented.

### Epistatic effects between *Pgc1α* and *Pck1*

**Fig 4:**
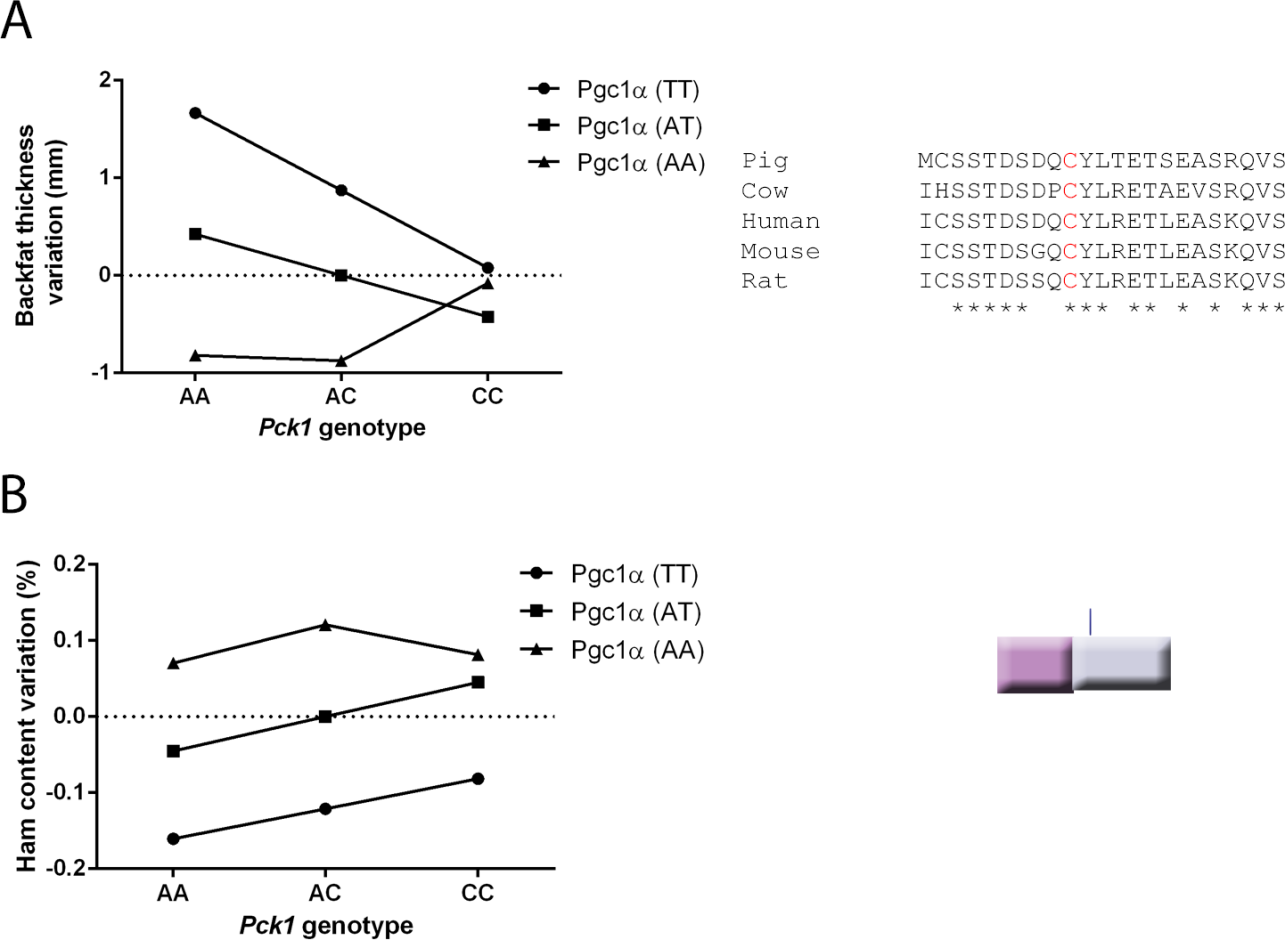
Epistasis between *Pg1α* c.A1378T and *Pck1* c.A2456C polymorphisms. Graphical representation of epistatic effects from data presented in Table 1 for traits (A) backfat thickness and (B) ham content. The additive effect of locus *Pgc1α* is greater in the Pck1_AA_ background and disappears (backfat thickness variation) or is diminished (ham content variation) in the Pck1_CC_ background.

The Bayesian statistical analysis also detects epistatic interactions between *Pgc1α* and *Pck1*. Table 1 also shows the results of this analysis on conformational and meat quality traits as in the former section; it shows only epistatic effects with a posterior probability above 0.9 or below 0.1. (S1 Table shows the lack of appreciable epistatic effects on compositional traits). The interaction between *Pgc1α* and *Pck1* (see Table 1) showed a high number (11/19) of these above-threshold interactions, that is almost 60% of total epistatic events. These interactions have some internal coherence: the three exudation traits show the same trend, being increased by the combination of Pck1 c.2456C and Pgc1α c.1378A alleles whereas the same combination decreased the adipose tissue content for four traits (backfat thickness, belly, lard and adipose tissue content in ham). Fig 4 shows graphical representations of these epistatic effects for two traits: variation in the percentage of ham and variation in backfat thickness. In both cases, there is a clear additive effect associated with *Pcg1α* for genotypes AA and AC at *Pck1* locus, but it disappears (backfat thickness) or is diminished (ham content) for individuals with genotype CC.

## Discussion

PGC1α regulates the expression of gluconeogenic enzymes [4,5,13] such as PCK1, an enzyme which is in a crossroad of several metabolic pathways [2]. PCK1 expression is regulated, among others, by PPARγ [17] which is activated by PGC1α [1]. We report here how alterations in the interaction between the transcription factors PGC1α and PPARγ caused by different levels of post-translational glycosylation of genetic variants of the former might be responsible for differences in phenotypic traits related to PCK1 activity. Furthermore, these molecular mechanisms can explain, at least in part, the apparent epistatic effects observed between PGC1α and PCK1.

Pig Pgc1α has 95% identity and 98.2% similarity with human PGC1α, which justified using a gluconeogenic human cell line with high transfection efficiency, HEK293T, to study at the molecular level the genetic interaction between PGC1α and PCK1, focusing on a common Pgc1α polymorphism found in pigs, a C430S substitution. PCK1 mRNA levels strongly increased when cells overexpressed each Pgc1α variant but that effect was higher (2.4 fold) when overexpressing Pgc1α WT (Fig 1B). In the same way, when overexpressing Pgc1α WT, cells reached higher PCK1 protein levels (20%) (Fig 1C and 1D) and produced more glucose (19%) (Fig 1E). Therefore, our results indicate that these two Pgc1α variants have different transcriptional activities with respect to PCK1 expression.

In order to identify the molecular mechanism underlying the differential PCK1 expression mediated by Pgc1α variants, we first analyzed their stability in HEK293T cells. To our surprise, the most active variant with respect to PCK1 expression, Pgc1α WT, was less stable than Pgc1α p.C430S. Zhang et al. [19] described the presence of a splicing isoform of PGC1α (NT-PGC1α) that was mainly present in the cytosol and had increased half-life in the cell [20]. Altered subcellular localization could therefore explain the differences we observed. However, our results showed that this was not the case since both variants were clearly located in the nucleus (Fig 2B).

Since the ability of Pgc1α WT to stimulate PCK1 synthesis was higher in spite of its lower stability, we decided to analyze whether differences in post-translational modifications (PTMs) could be responsible for the observed effects. We focused on the most common PTMs in PGC1α: acetylation, phosphorylation and O-GlcNAcylation [21]. Under standard culture conditions, no differences were found for any of these PTMs (Fig 3A) between Pgc1α variants. Furthermore, no signal was detected in the case of O-GlcNAcylation. Taking into account the difficulties in detecting O-GlcNAcylation in PGC1α [16], we co-transfected HEK293T cells with human O-GlcNAc transferase (OGT) with either Pgc1α p.C430S or Pgc1α WT to ensure more OGT activity in the cells. On the other hand, transfected HEK293T cells were treated with PUGNAc, an O-GlcNAcase inhibitor, as previously described [16]. Under these conditions, neither acetylation nor phosphorylation were affected. Nevertheless, Pgc1α p.C430S showed not only higher levels of O-GlcNAcylation (Fig 3B and 3C), but also its interaction with PPARγ was considerably weakened (Fig 3B). Acetylation of some residues has been described to activate [22] or to inactivate [23] PGC1α. The same occurs with phosphorylation [24,25]. In both cases, the detected signals were weak. In the case of O-GlcNAcylation, it has been described to stabilize and to increase PGC1α activity via OGT/HIF1 complex [16]. If this is true, the increased O-GlcNAcylation of p.C430S should contribute to the increased stability of this protein (Fig 2A) and, thus, should facilitate the interaction of this variant of Pgc1α with other proteins. However, we found exactly the opposite: a significantly weaker interaction of Pgc1α p.C430S with PPARγ than that for the WT variant. As this interaction is the one that ultimately leads to increased PCK1 expression, we could reasonably conclude that increased levels of O-GlcNAcylation impair the PPARγ-Pgc1α interaction and explain the higher PCK1 expression upon overexpression of Pgc1α WT (Fig 1B).

The existence of gene variability defines phenotypic differences between individuals. In most cases, the individual effects of these genes are subtle but not negligible. Quantitative traits play a relevant role in animal [26] and plant [27] breeding, tissue development [28] and disease [29]. Part of these effects can be a consequence of the existence of epistatic interactions [30]. The importance of *Pck1* for pig breeders has been highlighted by our recent report: the existence of a SNP in the coding region of this gene which resulted in the expression of an enzyme with substantially modified biochemical properties associated with better meat quality traits [3]. Given the importance of quantitative traits and the existence of variability in both pig *Pgc1α* and *Pck1*, we studied the epistatic interactions between these two genes and the potential mechanisms underlying this effect, focusing on the *Pgc1α* c.T1378A (p.C430S) polymorphism described in this work and on the Pck1 c.A2456C one (p.M139L) described in our former work [3].

We first evaluated *Pgc1α* additive effects on a Duroc x Landrace/Large White population and we found relevant association in just 6 out of 55 traits (noble parts yield, tenderloin content, protein content in loin, protein content in *B*. *femoris*, moisture in *B*. *femoris* and moisture in ham; S1 Table). This is even less than what could be expected just by chance. Liu et al. [12] described the effects of pig *Pgc1α* c.T1378A substitution on a Tibetan breed population and found significant effects on IMF content and muscle fiber size. The effects on IMF can be related to the expression of metabolic genes such as *Pck1*. However, although PGC1α is involved in defining muscle fiber type [6], it has no effects on fiber size in mice [31]. The fact that we did not find differences in similar traits in our pig population may be either a consequence of the low frequency of the *Pgc1α* TT genotype (14.1%) in our pigs compared to that of the Tibetan breeds studied (63.9%) by Liu et al [12] or to breed-specific effects as has been suggested by other authors [32].

The lack of enough animals representing all the possible haplotypes precluded to extend the study to additive x dominant, dominant x additive and dominant x dominant interactions. Nevertheless, our epistatic analysis of additive x additive interactions under the Bayesian approach showed a total of almost 60% of total epistatic interactions with Bayesian posterior probabilities above 0.9 or below 0.1 (Table 1). This percentage should be compared with the expected 20% of Bayesian posterior probabilities over 0.9 or below 0.1 that can be expected just by chance. Moreover, these relevant posterior probabilities are found in traits mostly related to percentage of adipose tissue content and meat water holding capacity. These traits were also the most affected by the *Pck1* polymorphism [3]. The combination of Pgc1α p.C430S and Pck1 p.M139L was associated to diverse effects. On one side, Pgc1α p.C430S reinforced the effects of Pck1 p.M139L decreasing meat quality, that is, yielding higher drip loss and exudation. On the other side, this very same allele combination is associated to lower backfat thickness and lower percentages of cuts rich in adipose tissue (lard and belly). The latter would be more convenient from an economical point of view because these cuts are less valued. Therefore, there is no single combination of *Pgc1α* and *Pck1* alleles with only positive effects on both meat quality and carcass conformation traits as happened with *Pck1* [3]. This comes to no surprise because PGC1α could produce its effects not only through PCK1 but through many other protein pathways [4,5]. Thus, the results of the epistatic analysis indicate that the application of these polymorphisms in marker-assisted selection must be taken with caution because, 1) there is not a clear optimum genotypic configuration and 2) it is plausible that the detected epistatic interaction is part of a more complex epistatic network with unpredictable results with the available information.

In conclusion, we report that differential O-GlcNAcylation of Pgc1α regulates PCK1 activity and this molecular mechanism could explain at least in part the epistatic interaction between both genes. This should be taken into account if these genes are used for animal selection in pig breeding.

## Supporting information

S1 Table Posterior mean estimate and Bayesian posterior probability of all analyzed traits. (XLSX)

## References

[1] Puigserver P, Wu Z, Park CW, Graves R, Wright M, Spiegelman BM. A cold-inducible coactivator of nuclear receptors linked to adaptive thermogenesis. Cell. 1998;92: 829–839. doi: 10.1016/S0092-8674(00)81410-5 9529258 10.1016/s0092-8674(00)81410-5

[2] Yang J, Kalhan SC, Hanson RW. What is the metabolic role of phosphoenolpyruvate carboxykinase? Journal of Biological Chemistry. 2009 pp. 27025–27029. doi: 10.1074/jbc.R109.040543 19636077 10.1074/jbc.R109.040543 PMC2785631

[3] Latorre P, Burgos C, Hidalgo J, Varona L, Carrodeguas JA, López-Buesa P. c.A2456C-substitution in Pck1 changes the enzyme kinetic and functional properties modifying fat distribution in pigs. Sci Rep. 2016;6: 19617 doi: 10.1038/srep19617 26792594 10.1038/srep19617 PMC4726144

[4] Fernandez-Marcos PJ, Auwerx J. Regulation of PGC-1α, a nodal regulator of mitochondrial biogenesis. American Journal of Clinical Nutrition. 2011 doi: 10.3945/ajcn.110.001917 21289221 10.3945/ajcn.110.001917 PMC3057551

[5] Finck BN, Kelly DP. PGC-1 coactivators: Inducible regulators of energy metabolism in health and disease. Journal of Clinical Investigation. 2006 pp. 615–622. doi: 10.1172/JCI27794 16511594 10.1172/JCI27794 PMC1386111

[6] Lin J, Wu H, Tarr PT, Zhang C-Y, Wu Z, Boss O, et al Transcriptional co-activator PGC-1 alpha drives the formation of slow-twitch muscle fibres. Nature. 2002;418: 797–801. doi: 10.1038/nature00904 12181572 10.1038/nature00904

[7] Chen Y, Mu P, He S, Tang X, Guo X, Li H, et al Gly482Ser mutation impairs the effects of peroxisome proliferator-activated receptorα coactivator-1α on decreasing fat deposition and stimulating phosphoenolpyruvate carboxykinase expression in hepatocytes. Nutr Res. 2013;33: 332–339. doi: 10.1016/j.nutres.2013.02.003 23602251 10.1016/j.nutres.2013.02.003

[8] Kunej T, Wu XL, Berlic TM, Michal JJ, Jiang Z, Dovc P. Frequency distribution of a Cys430Ser polymorphism in peroxisome proliferator-activated receptor-gamma coactivator-1 (PPARGC1) gene sequence in Chinese and Western pig breeds. J Anim Breed Genet. 2005;122: 7–11. doi: 10.1111/j.1439-0388.2004.00498.x 16130483 10.1111/j.1439-0388.2004.00498.x

[9] Erkens T, Rohrer GA, Van Zeveren A, Peelman LJ. SNP detection in the porcine PPARGC1A promoter region and 3’UTR, and an association study in a Landrace-Duroc-Large White population. Czech J Anim Sci. 2009;54: 408–416.

[10] Erkens T, De Smet S, Van den Maagdenberg K, Stinckens A, Buys N, Van Zeveren A, et al Association analysis of PPARGC1A mutations with meat quality parameters in a commercial hybrid pig population. Czech J Anim Sci. 2010;55: 200–208.

[11] Gandolfi G, Cinar MU, Ponsuksili S, Wimmers K, Tesfaye D, Looft C, et al Association of PPARGC1A and CAPNS1 gene polymorphisms and expression with meat quality traits in pigs. Meat Sci. 2011;54: 478–485. doi: 10.1016/j.meatsci.2011.05.015 21680104 10.1016/j.meatsci.2011.05.015

[12] Liu R, Li J, Lv X. Association of PGC-1α Gene with Intramuscular Fat Content and Muscle Fiber Traits and Gene Expression in Tibetan Pigs. J Anim Vet Adv. 2011;10: 2301–2304.

[13] Yoon JC, Puigserver P, Chen G, Donovan J, Wu Z, Rhee J, et al Control of hepatic gluconeogenesis through the transcriptional coactivator PGC-1. Nature. 2001;413: 131–138. doi: 10.1038/35093050 11557972 10.1038/35093050

[14] Alvarez-Castro JM, Carlborg O. A Unified Model for Functional and Statistical Epistasis and Its Application in Quantitative Trait Loci Analysis. Genetics. 2007;176: 1151–1167. doi: 10.1534/genetics.106.067348 17409082 10.1534/genetics.106.067348 PMC1894581

[15] Gelfand A, Smith AFM. Sampling-Based Approaches to Calculating Marginal Densities. J Am Stat Assoc. 1990;85: 398–409. doi: 10.2307/2289776

[16] Bin Ruan H, Han X, Li MD, Singh JP, Qian K, Azarhoush S, et al O-GlcNAc transferase/host cell factor C1 complex regulates gluconeogenesis by modulating PGC-1α stability. Cell Metab. 2012;16: 226–237. doi: 10.1016/j.cmet.2012.07.006 22883232 10.1016/j.cmet.2012.07.006 PMC3480732

[17] Tontonoz P, Hu E, Devine J, Beale EG, Spiegelman BM. PPAR gamma 2 regulates adipose expression of the phosphoenolpyruvate carboxykinase gene. Mol Cell Biol. 1995;15: 351–357. doi: 10.1128/MCB.15.1.351 7799943 10.1128/mcb.15.1.351 PMC231968

[18] Glorian M, Duplus E, Beale EG, Scott DK, Granner DK, Forest C. A single element in the phosphoenolpyruvate carboxykinase gene mediates thiazolidinedione action specifically in adipocytes. Biochimie. 2001;83: 933–943. doi: 10.1016/S0300-9084(01)01343-8 11728630 10.1016/s0300-9084(01)01343-8

[19] Zhang Y, Huypens P, Adamson AW, Chang JS, Henagan TM, Boudreau A, et al Alternative mRNA splicing produces a novel biologically active short isoform of PGC-1α. J Biol Chem. 2009;284: 32813–32826. doi: 10.1074/jbc.M109.037556 19773550 10.1074/jbc.M109.037556 PMC2781698

[20] Trausch-Azar J, Leone TC, Kelly DP, Schwartz AL. Ubiquitin Proteasome-dependent Degradation of the Transcriptional Coactivator PGC-1α via the N-terminal Pathway. J Biol Chem. 2010;285: 40192–40200. doi: 10.1074/jbc.M110.131615 20713359 10.1074/jbc.M110.131615 PMC3001001

[21] Housley MP, Udeshi ND, Rodgers JT, Shabanowitz J, Puigserver P, Hunt DF, et al A PGC-1alpha-O-GlcNAc transferase complex regulates FoxO transcription factor activity in response to glucose. J Biol Chem. 2009;284: 5148–5157. doi: 10.1074/jbc.M808890200 19103600 10.1074/jbc.M808890200 PMC2643526

[22] Higashida K, Kim SH, Jung SR, Asaka M, Holloszy JO, Han DH. Effects of Resveratrol and SIRT1 on PGC-1alpha Activity and Mitochondrial Biogenesis: A Reevaluation. PLoS Biol. 2013;11: e1001603 doi: 10.1371/journal.pbio.1001603 23874150 10.1371/journal.pbio.1001603 PMC3706311

[23] Lagouge M, Argmann C, Gerhart-Hines Z, Meziane H, Lerin C, Daussin F, et al Resveratrol Improves Mitochondrial Function and Protects against Metabolic Disease by Activating SIRT1 and PGC-1α. Cell. 2006;127: 1109–1122. doi: 10.1016/j.cell.2006.11.013 17112576 10.1016/j.cell.2006.11.013

[24] Jäger SS, Handschin CC, St-Pierre JJ, Spiegelman BMBM. AMP-activated protein kinase (AMPK) action in skeletal muscle via direct phosphorylation of PGC-1alpha. Pnas. 2007;104: 12017–12022. doi: 10.1073/pnas.0705070104 17609368 10.1073/pnas.0705070104 PMC1924552

[25] Li X, Monks B, Ge Q, Birnbaum MJ. Akt/PKB regulates hepatic metabolism by directly inhibiting PGC-1alpha transcription coactivator. Nature. 2007;447: 1012–1016. doi: 10.1038/nature05861 17554339 10.1038/nature05861

[26] Dekkers J. Application of Genomics Tools to Animal Breeding. Curr Genomics. 2012;13: 207–212. doi: 10.2174/138920212800543057 23115522 10.2174/138920212800543057 PMC3382275

[27] St.Clair DA. Quantitative Disease Resistance and Quantitative Resistance Loci in Breeding. Annu Rev Phytopathol. 2010;48: 247–268. doi: 10.1146/annurev-phyto-080508-081904 19400646 10.1146/annurev-phyto-080508-081904

[28] Wood AR, Esko T, Yang J, Vedantam S, Pers TH, Gustafsson S, et al Defining the role of common variation in the genomic and biological architecture of adult human height. Nat Genet. 2014;46: 1173–86. doi: 10.1038/ng.3097 25282103 10.1038/ng.3097 PMC4250049

[29] Plomin R, Haworth CM a, Davis OSP. Quantitative Traits [Internet]. Genetics. 2009 doi: 10.1038/nrg2670 10.1038/nrg2670 19859063

[30] Mackay TFC. Epistasis and quantitative traits: using model organisms to study gene-gene interactions. Nat Rev Genet. 2013;15: 22–33. doi: 10.1038/nrg3627 24296533 10.1038/nrg3627 PMC3918431

[31] Takikita S, Schreiner C, Baum R, Xie T, Ralston E, Plotz PH, et al Fiber type conversion by PGC-1α. activates lysosomal and autophagosomal biogenesis in both unaffected and pompe skeletal muscle. PLoS One. 2010;5 doi: 10.1371/journal.pone.0015239 21179212 10.1371/journal.pone.0015239 PMC3001465

[32] Wood JD, Nute GR, Richardson RI, Whittington FM, Southwood O, Plastow G, et al Effects of breed, diet and muscle on fat deposition and eating quality in pigs. Meat Sci. 2004;67: 651–667. doi: 10.1016/j.meatsci.2004.01.007 22061815 10.1016/j.meatsci.2004.01.007

