# Correction: Asymptomatic aortic stenosis: An assessment of patients' and of their general practitioners' knowledge, after an indexed specialized assessment in community practice

**DOI:** 10.1371/journal.pone.0183566

**Published:** 2017-08-15

**Authors:** Raphaëlle-Ashley Guerbaai, Gabriel Fustier, Pierre-Vladimir Ennezat, Anne Ringle, Camille Trouillet, Pierre Graux, André Vincentelli, Christophe Tribouilloy, Sylvestre Maréchaux

The first author's name is spelled incorrectly. The correct name is: Raphaëlle-Ashley Guerbaai. The correct citation is: Guerbaai R-A, Fustier G, Ennezat P-V, Ringle A, Trouillet C, Graux P, et al. (2017) Asymptomatic aortic stenosis: An assessment of patients’ and of their general practitioners’ knowledge, after an indexed specialized assessment in community practice. PLoS ONE 12(6): e0178932. https://doi.org/10.1371/journal.pone.0178932
