# Correction: O-GlcNAcylation mediates the control of cytosolic phosphoenolpyruvate carboxykinase activity via *Pgc1α*

**DOI:** 10.1371/journal.pone.0183735

**Published:** 2017-08-17

**Authors:** The PLOS ONE Staff

Fig 4 appears incorrectly. Please see the complete, correct Fig 4 here.

**Fig 4:**
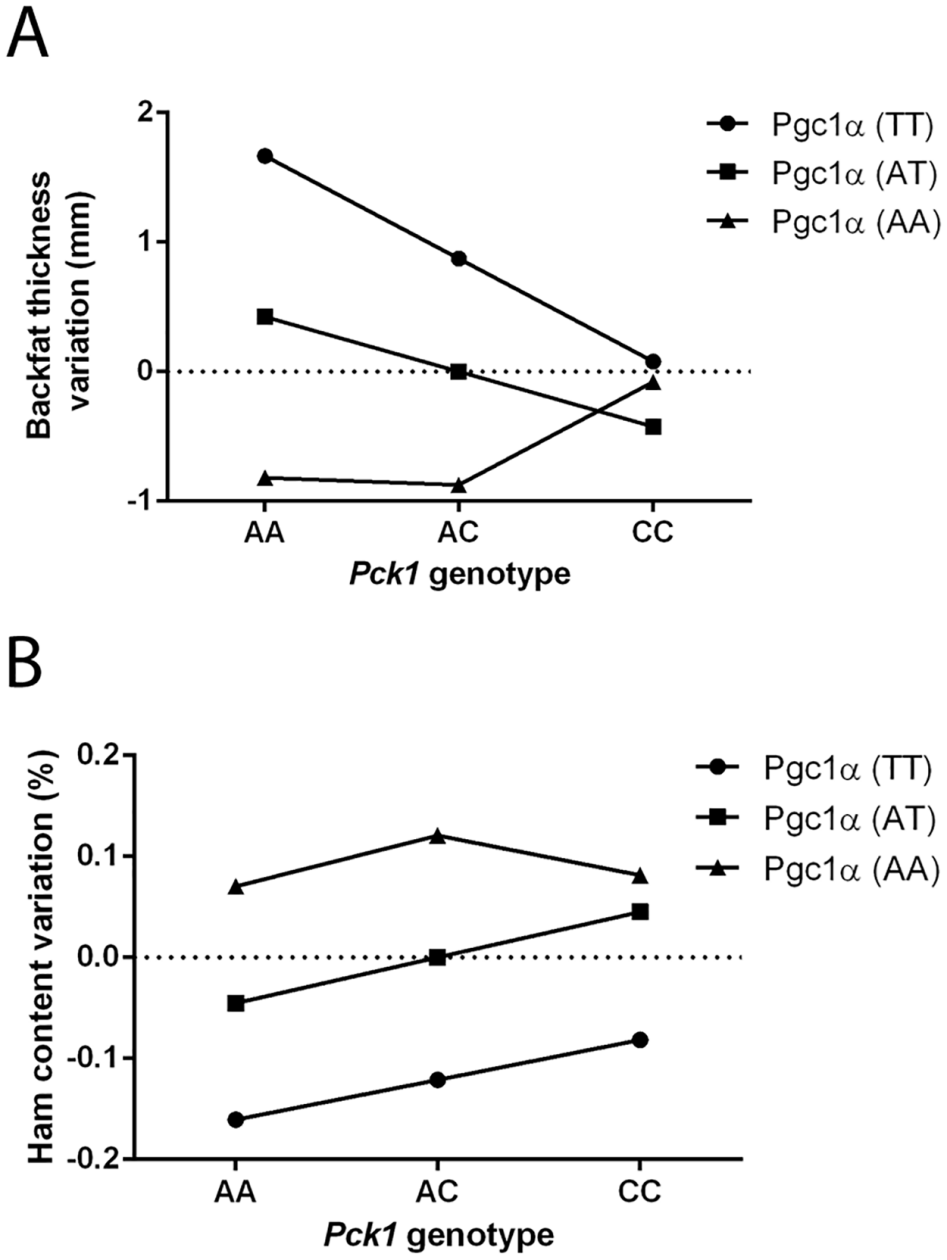
Epistasis between Pg1α c.A1378T and Pck1 c.A2456C polymorphisms. Graphical representation of epistatic effects from data presented in Table 1 for traits (A) backfat thickness and (B) ham content. The additive effect of locus Pgc1α is greater in the Pck1AA background and disappears (backfat thickness variation) or is diminished (ham content variation) in the Pck1CC background.

The publisher apologizes for the error.
